# Expression of matrix metalloproteinases in cerebral amyloid angiopathy-a systematic review

**DOI:** 10.3389/fneur.2026.1753708

**Published:** 2026-02-06

**Authors:** Hanying Gu, Xiuxia Shi, Jiangtao Zhang

**Affiliations:** 1 Emergency Department, Chengde Medical College, Chengde, China; 2 Department of Critical Care Medicine, Chengde Central Hospital, Chengde, China; 3 Department of Neurology, Chengde Central Hospital, Chengde, China

**Keywords:** biomarkers, cerebral amyloid angiopathy, hemorrhage, inhibitors, intracerebral, matrix metalloproteinases

## Abstract

**Objective:**

This study aimed to conduct a systematic review of the expression levels of matrix metalloproteinases (MMPs) and tissue inhibitors of metalloproteinases (TIMPs) in cerebral amyloid angiopathy (CAA).

**Methods:**

This systematic review was conducted in accordance with the Preferred Reporting Items for Systematic Reviews and Meta-Analyses (PRISMA) guidelines. The PubMed, Embase, and Web of Science databases were searched to identify relevant studies. Two researchers independently screened the literature, extracted data, and assessed the study quality.

**Results:**

Five studies evaluating a total of 442 participants were included. The findings revealed dysregulation of the MMP/TIMP system in the cerebral blood vessels of patients with CAA. Specifically, in comparison with patients without CAA, those with CAA showed significantly upregulated expression of TIMP-3 and TIMP-4 in the cerebral blood vessels, and TIMP-4 levels were positively correlated with the severity of CAA. MMP-9 expression in patients with CAA-related intracerebral hemorrhage (CAA-ICH) was significantly higher than in those without hemorrhage, while TIMP-3 expression was lower in patients with CAA-ICH; these findings suggest that an imbalance between MMP-9 and TIMP-3 may increase the risk of hemorrhage. Cerebrospinal fluid (CSF) and serum biomarker studies showed that patients with CAA had decreased TIMP-4 levels in the CSF and significantly lower serum MMP-2 levels.

**Conclusion:**

The findings of this study indicated an imbalance in the MMP/TIMP system in CAA, which may be involved in its vascular pathological mechanism. However, the existing evidence is insufficient to support the use of MMPs/TIMPs as reliable biomarkers for CAA. Therefore, further evaluation of their diagnostic and therapeutic value is required in future studies.

**Systematic review registration:**

This systematic review was registered in PROSPERO (Unique Identifier: CRD420251230405). The protocol can be accessed at: https://www.crd.york.ac.uk/PROSPERO/view/CRD420251230405, CRD420251230405.

## 1 Introduction

Cerebral amyloid angiopathy (CAA), an age-related form of cerebral small-vessel disorder, is characterized by progressive deposition of *β*-amyloid protein (Aβ) within the walls of small arteries and capillaries in the pia mater and cortex (1, 2). CAA is most commonly observed in older adults, and its prevalence in patients with Alzheimer’s disease (AD) is as high as 80–90% (3, 4). Although both CAA and AD share Aβ as a pathological factor, they show differences in terms of deposition sites and clinical manifestations, leading to a complex pathological relationship that can be summarized as “one peptide, two pathways” (5). The key clinical and imaging features of CAA are provided in Table 1 (6–8). The diagnosis of CAA is primarily based on the Boston criteria 2.0 (9, 10); however, no effective treatments are available for this disease.

**Table 1:** Major clinical and imaging features of cerebral amyloid angiopathy (CAA) (6–8).

Characteristic type	Specific manifestations
Clinical features	CAA-ICH, progressive cognitive impairment, TFNE, headache/epilepsy
Imaging markers	CMBs, cSS, WMH, cSAH
Pathological features	Aβ deposition in the vascular wall, loss of vascular smooth muscle cells, fibrosis of the vascular wall
High-risk factors	ApoEε4 allele, advanced age, comorbidity of Alzheimer’s disease

CAA, Cerebral amyloid angiopathy; CAA-ICH, Cerebral amyloid angiopathy with intracerebral hemorrhage; CMB, Cerebral microbleed; cSAH, Convexity subarachnoid hemorrhage; cSS, Cortical superficial siderosis; TFNE, Transient focal neurologic episode; WMH, White matter hyperintensity.

Matrix metalloproteinases (MMPs) are a family of zinc-dependent endopeptidases capable of degrading almost all components of the extracellular matrix (ECM), and they play central roles in various physiological and pathological processes, including tissue remodeling, angiogenesis, and inflammatory responses (11, 12). MMP activity is strictly regulated by tissue inhibitors of metalloproteinases (TIMPs) (13, 14). Physiological conditions are characterized by a delicate equilibrium between MMPs and TIMPs. However, under pathological conditions, including cerebral ischemia, carotid atherosclerotic plaques, arteriovenous malformations, and aneurysms, this equilibrium is disrupted, causing increased MMP expression and proteolytic activity and ultimately resulting in excessive ECM degradation, tissue structural damage, and functional impairment (15, 16).

In cerebrovascular diseases, excessive MMP activation has been linked to blood–brain barrier (BBB) disruption, neuroinflammation, and secondary injury following intracerebral hemorrhage (ICH) (17, 18). Since the core pathology of CAA involves compromised structural integrity of the vascular wall, an imbalance in the MMP/TIMP system may play a crucial role in its pathogenesis (19). For example, excessive MMP activity may degrade collagen and laminin in the vascular basement membrane, causing thinning of the blood vessel wall, decreased elasticity, and rupture and bleeding during blood-pressure fluctuations (20, 21). These findings were first demonstrated in a preclinical study that identified MMP activation as a downstream executor of the vascular damage caused by CAA-associated pathological factors (such as activated platelets), and showed that vascular integrity can be effectively preserved by inhibiting MMP activity (22). Several recent studies have also focused on the changes in MMP and TIMP expression in CAA and their relationship with clinical manifestations. In particular, since Aβ serves as a shared pathological factor for CAA and AD, studies are required to determine whether the observed dysregulation of MMPs/TIMPs can be precisely attributed to cerebrovascular Aβ deposition (i.e., CAA pathology), parenchymal Aβ plaques (as in AD), or both. Unraveling this relationship is key to understanding the specificity of MMP/TIMP alterations in CAA and developing targeted therapeutic strategies.

The ongoing development of matrix metalloproteinase inhibitors (MMPIs) initially focused on antitumor therapy (23, 24). However, with a deeper understanding of the complexity and specificity of the function of MMPs, highly selective MMPIs targeting CAA may also be developed (25, 26). Tetracycline antibiotics, including doxycycline and minocycline, have also been found to act as nonantibiotic-dependent MMPIs (27, 28).

Against this research background, we aimed to systematically review and integrate the findings of existing studies on MMPs in CAA to gain a deeper understanding of the pathogenesis and assess the potential value of MMPs as biomarkers or therapeutic targets.

## 2 Methods

This systematic review adhered to the Preferred Reporting Items for Systematic Reviews and Meta-Analyses (PRISMA) guidelines (20, 29) and was prospectively registered on the PROSPERO platform on November 19, 2025 (CRD420251230405). Since this study analyzes previously published data, ethical review is not required.

### 2.1 Search strategy

We systematically searched the PubMed, Embase, and Web of Science databases, covering the period from database inception to September 2025. The search strategy incorporated both controlled vocabulary (e.g., MeSH terms) and free-text terms, including “Cerebral Amyloid Angiopathy,” “Matrix Metalloproteinase,” “MMPs,” and “Matrixin.” The complete search strategy is provided in Supplementary material S1.

### 2.2 Inclusion and exclusion criteria

After the initial search, studies were systematically screened against predefined inclusion criteria by reviewing titles and abstracts. The inclusion criteria were as follows: (1) Studies including patients with a high probability of CAA who were diagnosed on the basis of pathological findings or met the Boston criteria; (2) studies evaluating the expression levels of MMPs, TIMPs, and MMPs/TIMPs in brain tissue, cerebrospinal fluid (CSF) or serum; and (3) observational studies (case–control, cohort studies, or cross-sectional studies). Studies meeting any of the following criteria were excluded: (1) studies with non-CAA study participants (e.g., AD patients alone), (2) animal experiments, (3) studies with full text unavailable or incomplete data, (4) duplicate publications (the study with the most complete data was selected), (5) reviews, case reports, case series, commentaries, conference abstracts.

### 2.3 Study screening and data extraction

Literature management and duplicate removal were performed using EndNote 20 software. Records. The study screening process was conducted independently by two researchers (HG and XS). Initial screening was performed by reading the titles and abstracts to exclude studies that clearly did not meet the inclusion criteria. The remaining studies were then read in full, and a final screening was conducted on the basis of the inclusion and exclusion criteria. Any disagreements were resolved through discussion with a third researcher (JZ).

Data extraction was performed using a predefined data-extraction form. The extracted data included information regarding first author, publication year, country of study, study type, basic information of study participants (age, sex, sample size), sample type (brain tissue, CSF, blood), detection method, main MMP types, expression results, and correlation with clinical/pathological indicators. Data extraction was also completed independently by the two researchers (HG and XS) and cross-checked to ensure accuracy. Conflicts were resolved through discussion with the third researcher (JZ).

### 2.4 Quality assessment

The methodological quality of the included studies was assessed, and relevant data and information were compiled and extracted. This study was completed independently by the two researchers (HG and XS) in accordance with the inclusion and exclusion criteria for the literature. Disagreements were discussed and resolved by consensus or by a third researcher (JZ). The quality of the included studies was assessed using the Newcastle Ottawa Scale (NOS). Since all the literature included in this study was observational, the NOS scale was used for scoring (NOS). Since all the literature included in this study was observational, the NOS scale was used for scoring (30). The total NOS score was 9 points, with 1 point for studies marked with “*,” 4 points for the choice of study participants, 2 points for intergroup comparability, and 3 points for outcome measurement. A score of 7–9 was defined as high quality, 4–6 as moderate quality, and 0–3 as low quality.

### 2.5 Data analysis

Given the anticipated substantial clinical and methodological heterogeneity regarding design, population, intervention, and outcome measurement in the included studies, we planned to conduct a qualitative narrative synthesis rather than a quantitative meta-analysis. We categorized and summarized the results on the basis of the research themes, mainly covering two aspects: (1) the expression and role of MMPs/TIMPs in CAA brain tissue; and (2) the potential of MMPs/TIMPs as humoral biomarkers for CAA.

## 3 Results

### 3.1 Study screening

**Figure 1:**
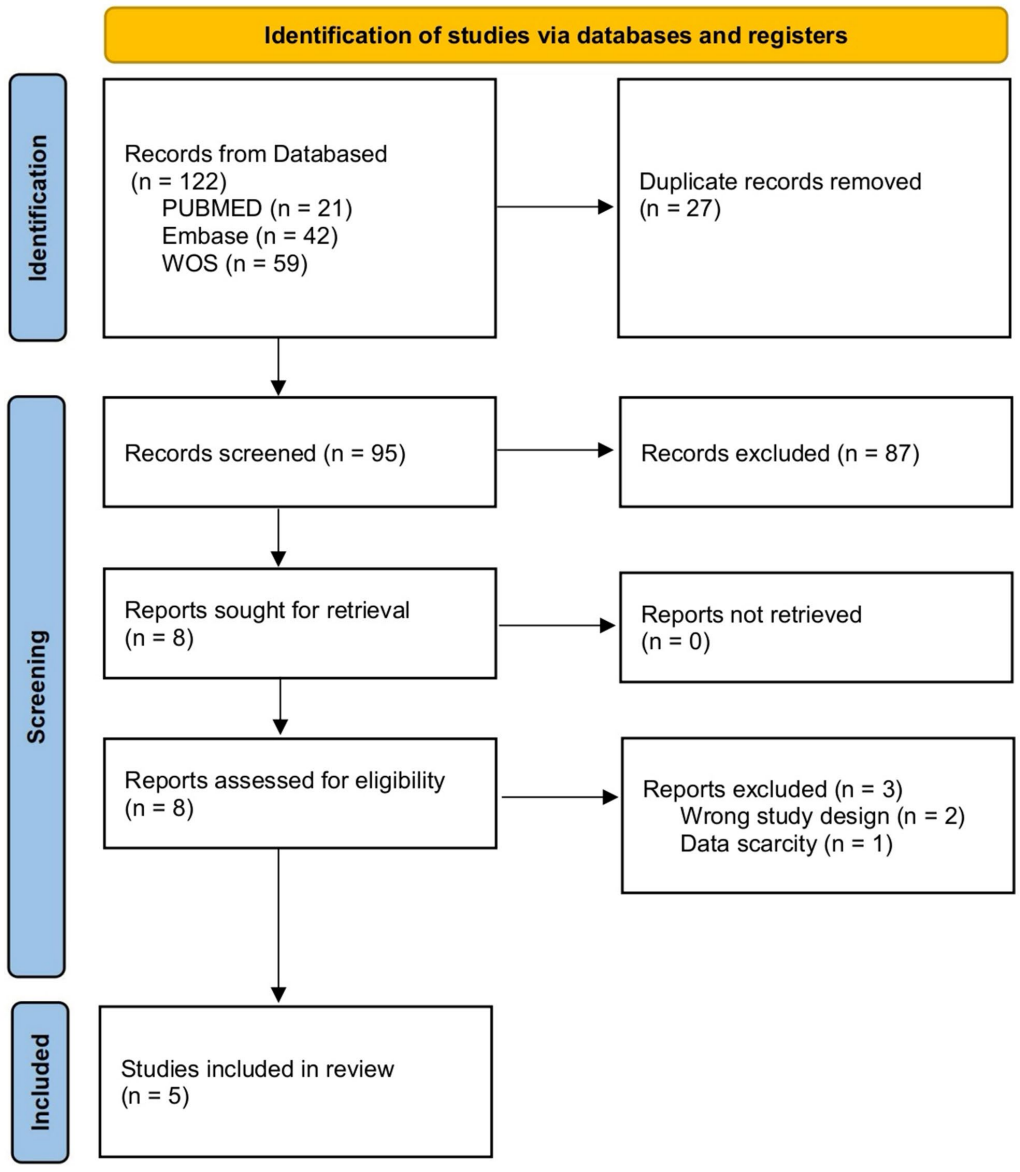
Flow diagram of the selection process.

The initial database searches identified 122 potentially relevant records. After removing duplicates, 95 articles remained. After title and abstract screening, 87 clearly irrelevant records were excluded. The remaining eight articles underwent full-text evaluation. Two studies were excluded since they included non-CAA study populations, and one was excluded due to incomplete information. Ultimately, five studies met the inclusion criteria and were included in this systematic review for qualitative analysis. Figure 1 shows the PRISMA flow diagram of study selection.

### 3.2 Characteristics and quality assessment of the included studies

All five included studies were observational in design, encompassing a total of 442 participants. The detailed summary characteristics of the included studies are shown in Table 2 (31–35).

**Table 2:** Characteristics of the included studies.

Author/Year	Country	Study design	Intervention/control (n)	Sample	Outcome	Result
Jäkel et al. (2024) (31)	Nederland	Cross-sectional study	CAA (*n* = 57/38) vs. controls (*n* = 42/37)	Brain tissue, CSF, and serum	TIMP-4	In comparison with the findings in the control group, TIMP-4 expression in the cerebral blood vessels of CAA patients increased and was correlated with the severity of CAA; TIMP-4 levels in the CSF decreased and TIMP-4 levels in the serum increased.
Xia et al. (2021) (32)	China	Case–control study	CAA-ICH (*n* = 68) vs. healthy controls (*n* = 69)	Serum	MMP-2, MMP-3, and MMP-9	In comparison with the findings in the control group, the levels of MMP-2 in CAA-ICH patients decreased significantly, while those of MMP-9 significantly increased. MMP-3 levels showed no significant differences between the two groups.
Jäkel et al. (2020) (33)	Nederland	Case–control study	CAA-ICH (*n* = 11) vs. CAA-NH (*n* = 18) vs. Controls (*n* = 11)	Brain tissue	MMP-9, TIMP-3	In comparison with the findings in the control group, the MMP-2 level in patients with CAA-ICH significantly decreased, while the MMP-9 level in patients with CAA was significantly higher than that in patients without CAA. In comparison with patients showing CAA-NH, those showing CAA-ICH showed higher MMP-9 expression in the cerebral vessels and lower expression of TIMP-3.
Manousopoulou et al. (2017) (34)	UK	Cohort study	CAA (*n* = 7) vs. Controls (*n* = 7/5)	Brain tissue	TIMP-3	TIMP-3 expression and localization in brain tissue were significantly upregulated in the vascular walls of patients with CAA in comparison with young and elderly controls, and were co-localized with Aβ deposition.
Tanskanen et al. (2011) (35)	Finland	Cohort study	CAAH (15/36) vs. Controls (2/19)	Brain tissue	MMP-19, MMP-26	The correlation between MMP-19 and cerebral hemorrhage depended on the presence of CAA, while MMP-26 was associated with CAA but not with cerebral hemorrhage.

CAA, Cerebral amyloid angiopathy; CAA-ICH, Cerebral amyloid angiopathy with intracerebral hemorrhage; CAA-NH, Cerebral amyloid angiopathy without intracerebral hemorrhage; CSF, Cerebrospinal fluid; MMP, Matrix metalloproteinase; TIMP, Tissue inhibitor of metalloproteinase.

### 3.3 Quality assessment results

Five clinical observational studies were assessed using the NOS scale. The assessment indicated that four studies were of high quality and one was of moderate quality. In summary, the quality scores of the included studies were above 6, and the overall quality was medium to high (Supplementary Table S3). The main potential sources of bias in these studies were the generally small sample sizes and the heterogeneity of research methodologies (such as sample source and MMP detection technology).

### 3.4 Relationship between MMP expression and CAA

#### 3.4.1 Changes in the expression levels of MMPs/TIMPs in the brain tissue in patients with CAA

Four studies used immunohistochemical analyses to detect the expression of MMPs/TIMPs in the brain tissue of CAA patients. Tanskanen et al. first documented that MMP-19 and MMP-26 were expressed in the brain tissue of patients with CAA. MMP-19 expression was associated with CAA and hemorrhage, while MMP-26 expression was associated only with CAA (35). Using proteomics approaches, Manousopoulou et al. demonstrated that TIMP-3 was significantly upregulated in the vascular wall of CAA and co-localized with Aβ deposition (34). Jäkel et al. further established that TIMP-3 expression in the blood vessels of patients with CAA was higher than that in the control group, regardless of whether they had cerebral hemorrhage; MMP-9 expression in the blood vessels of patients with CAA-related ICH (CAA-ICH) was significantly higher than that in patients with CAA without ICH (CAA-NH), while TIMP-3 expression was relatively reduced, suggesting that MMP-9/TIMP-3 imbalance promotes cerebral hemorrhage (33). Jäkel et al. also found that TIMP-4 expression in the cerebral blood vessels of CAA was upregulated and positively correlated with the severity of CAA; the TIMP-4 level in patients with CAA-ICH was higher than that in patients with CAA-NH, suggesting that TIMP-4 participates in vascular remodeling and reflects lesion severity (Table 3) (31).

**Table 3:** Summary of MMP and TIMP expression in cerebrovascular and body fluid biomarkers in patients with CAA and CAA-ICH included in the study.

Source of the sample		CAA vs. non-CAA		CAA-ICH vs. CAA-NH	
		Brain tissue	CSF/Serum	Brain tissue	Serum
MMPs	MMP-2	N. A.	N. A.	N. A.	↓
	MMP-3	N. A.	N. A.	N. A.	N. A.
	MMP-9	↑	N. A.	N. A.	↑
TIMPs	TIMP-3	↑	N. A.	↓	N. A.
	TIMP-3	↑	↓/↑	N. A.	N. A.

CAA, Cerebral amyloid angiopathy; CAA-ICH, Cerebral amyloid angiopathy with intracerebral hemorrhage; CAA-NH, Cerebral amyloid angiopathy without hemorrhage; CSF, Cerebrospinal fluid; MMPs, Matrix metalloproteinases; N. A., Not assessed; TIMPs, Tissue inhibitors of metalloproteinases.

#### 3.4.2 Potential of MMPs/TIMPs as fluid biomarkers in CAA

Two studies examined the levels of MMPs/TIMPs in the CSF and serum of patients with CAA. Xia et al. reported that serum MMP-2 levels declined and MMP-9 levels increased in patients with CAA-ICH, and the MMP-3 level was associated with the number of cerebral microbleeds (32). Jäkel et al. reported that CSF TIMP-4 levels declined and serum TIMP-4 levels increased in patients with CAA, indicating that TIMP-4 regulation is complex and that reduced TIMP-4 levels in the CSF may have diagnostic value (Table 3) (31). Furthermore, Vervuurt et al. reported that the MMP-2/TIMP-2 and MMP-14/TIMP-2 ratios in the CSF of patients with sporadic CAA and hereditary CAA were reduced, implying that ratio indices can indicate the pathological state more efficiently than the levels of a single molecule (19). Sakai et al. reported that TIMP-2 levels were elevated in the CSF of patients with CAA-related inflammation (CAA-ri) in the acute phase and remained high after treatment. Moreover, TIMP-1 levels were elevated after treatment, implying that the TIMP system was activated in the inflammatory subtype (23).

## 4 Discussion

This systematic review synthesized evidence supporting the conclusion that MMP/TIMP system imbalances are the mechanism underlying CAA vascular lesions, and that targeting this system may provide new directions for effective diagnosis and treatment of CAA. Multiple studies have provided evidence that the MMP/TIMP balance is disrupted in the cerebral blood vessels of patients with CAA. Although upregulation of TIMP-3 and TIMP-4 may represent a compensatory response to increased MMP activity, it cannot completely inhibit the destructive effects of MMPs (31, 34). In CAA-ICH, upregulation of MMP-9 expression and relative downregulation of TIMP-3 expression may cause the degradation rate of the vascular basement membrane to exceed its repair capacity, thereby accelerating the risk of hemorrhage (33). This imbalance is not limited to brain tissue and manifests in alterations of humoral biomarkers. Moreover, in comparison with changes in the levels of individual molecules, reductions in the MMP/TIMP ratios (e.g., the MMP-2/TIMP-2 and MMP-14/TIMP-2 ratios) in CSF may more reliably reflect the pathological status of CAA. Therefore, reduced MMP/TIMP ratios (such as the MMP-2/TIMP-2 and MMP-14/TIMP-2 ratios) could serve as more dependable diagnostic biomarkers (19). Notably, TIMP-4 expression is upregulated in the brain tissue of patients with CAA but is downregulated in CSF and elevated in serum, indicating a complex regulatory mechanism. The decline in TIMP-4 expression in CSF may have diagnostic value; however, its exact significance and the underlying reasons for the difference in expression between brain tissue and CSF warrant further elucidation in future studies (31). By showing that MMP inhibition confers cerebrovascular protection against CAA-associated damage (22), the preclinical evidence provided a theoretical basis for exploring MMPIs as potential therapeutic agents for CAA. Future efforts should focus on developing highly selective inhibitors of key MMPs, such as MMP-9, and on evaluating the efficacy of existing drugs, including tetracyclines, in CAA models (27).

This systematic review had several limitations. First, an important consideration arising from this review was whether the observed MMP/TIMP dysregulation is a direct consequence of cerebrovascular Aβ deposition (CAA pathology) or if it is also influenced by concurrent parenchymal Aβ pathology, as commonly observed in AD. Most of the included studies focused on cohorts defined by the CAA criteria; however, given the high co-occurrence of CAA and AD, complete dissociation of vascular and parenchymal Aβ effects remains challenging. Second, a major limitation of this review was the small sample size of the included original studies, which diminished the statistical power of individual findings and weakened the robustness of the existing qualitative evidence base. Third, the large variations in the clinical manifestations and pathological backgrounds of the patients included in the study resulted in substantial heterogeneity, limiting the scope to perform quantitative pooled analysis. Finally, inconsistencies in CSF sample-collection methods and times may have influenced the results for measurement of protease expression levels.

On the basis of these findings, the following directions are recommended for future research. First, investigations using designs that explicitly compare pure CAA, pure AD, and mixed-pathology cases, together with spatially resolved molecular analyses, are required to elucidate the specific contribution of vascular Aβ to MMP/TIMP imbalances. This distinction is crucial for assigning biomarker changes and therapeutic targets specifically to CAA. Second, to develop highly selective inhibitors, future studies should aim to identify the MMP types that play key roles in CAA (36). Third, the optimal timing of early intervention requires exploration, and biomarkers should be used to guide treatment (16). Reliable biomarkers, including CSF and imaging techniques, may play an important role in this process (19, 31). Fourth, studies should focus on improving the permeability of drugs to the BBB by, for example, using delivery systems that target transferrin receptors (37). Finally, randomized double-blind placebo-controlled trials with long-term follow-up data are essential for assessing the effects of MMPIs on disease recurrence and long-term prognosis.

## 5 Conclusion

The studies reviewed in this paper indicate dysregulation of MMPs/TIMPs in CAA. Although MMP/TIMP levels in brain tissue and CSF show potential as biomarkers, the existing evidence is inadequate to support the clinical use of these parameters. Future studies with more refined designs, larger sample sizes, and clearer pathological stratification are required to clarify the underlying mechanisms and determine whether MMPs/TIMPs can serve as reliable diagnostic tools or therapeutic targets for CAA.

## Data Availability

The original contributions presented in the study are included in the article/Supplementary material, further inquiries can be directed to the corresponding author/s.
