# Supplementary material for: Expression of matrix metalloproteinases in cerebral amyloid angiopathy-a systematic review
Source: Front Neurol. 2026 Feb 6;17:1753708. doi: 10.3389/fneur.2026.1753708 (PMC12920219; doi:10.3389/fneur.2026.1753708)
Supplement: Supplementary file 1 [file Table_1.DOCX]

Supplementary Material

# Supplementary Tables

| Datebase | Search Terms | Search Field | Search Results |
| --- | --- | --- | --- |
| PubMed | #1 "Cerebral Amyloid Angiopathy"[Mesh]  #2 (cerebral amyloid angiopath*[Title/Abstract]) OR (cerebrovascular amyloid[Title/Abstract]) OR (congophilic angiopath*[Title/Abstract])  #3 #1 OR #2  #4 "Matrix Metalloproteinases"[Mesh]  #5 (Matrix Metalloproteinase*[Title/Abstract]) OR (MMP[Title/Abstract]) OR (Gelatinase[Title/Abstract]) OR (Stromelysin[Title/Abstract]) OR (Collagenase[Title/Abstract])  #6 #4 OR #5  #7 #3 AND #6 | Title/Abstract | 21 |
| Embase | 1. exp cerebral amyloid angiopathy/  2. (cerebral amyloid angiopath* or cerebrovascular amyloid or congophilic angiopath*).ab,ti.  3. 1 or 2  4. exp matrix metalloproteinase/  5. (Matrix Metalloproteinase* or MMP or Gelatinase or Stromelysin or Collagenase).ab,ti.  6. 4 or 5  7. 3 and 6 | All Fields | 42 |
| Web of Science | #1 TS=(“cerebral amyloid angiopathy” OR “cerebrovascular amyloid” OR “congophilic angiopathy”)  #2 TS=(“Matrix Metalloproteinase*” OR MMP OR Gelatinase OR Stromelysin OR Collagenase)  #3 #1 AND #2 | All Fields | 59 |

**Supplementary Table 1.** Search strategy.

| CAA | cerebral amyloid angiopathy |
| --- | --- |
| CSVD | cerebral small vessel disorder |
| Aβ | β-amyloid |
| AD | alzheimer's disease |
| ICH | intracerebral haemorrhages |
| CAA-ICH | cerebral amyloid angiopathy with intracerebral hemorrhage |
| TFNE | transient focal neurologic episode |
| CMBs | cerebral micro bleeds |
| cSS | cortical superficial siderosis |
| WMH | white matter hyprintensity |
| cSAH | convexity subarachnoid hemorrhage |
| MMPs | matrix metalloproteinases |
| ECM | extracellular matrix |
| TIMPs | tissue inhibitors of metalloproteinases |
| BBB | blood-brain barrier |
| MMPIs | MMP inhibitors |
| CSF | Cerebrospinal fluid |
| CAA-NH | cerebral amyloid angiopathy without hemorrhage |
| NOS | Newcastle ottawa scale |
| sCAA | Sporadic cerebral amyloid angiopathy |
| D-CAA | Dutch-cerebral amyloid angiopathy |
| CAA-ri | CAA-related inflammation |

**Supplementary Table 2.** Abbreviations.

| Number | Author | Year | Components of the NOS checklist | | | Total score |
| --- | --- | --- | --- | --- | --- | --- |
|  |  |  | Selection | Comparability | Exposure/Outcome |  |
| 1 | Jäkel et al. | 2024 | 4 | 2 | 3 | 9 |
| 2 | Xia et al. | 2021 | 4 | 2 | 3 | 9 |
| 3 | Jäkel et al. | 2020 | 3 | 1 | 3 | 7 |
| 4 | Manousopoulou et al. | 2017 | 3 | 1 | 2 | 6 |
| 5 | Tanskanen et al. | 2011 | 4 | 1 | 3 | 8 |

**Supplementary Table 3.** The results of the NOS for assessment of the quality for the observational studies
